# Synthesis and Biological Evaluation of Achiral Indole-Substituted Titanocene Dichloride Derivatives

**DOI:** 10.1155/2012/905981

**Published:** 2012-06-12

**Authors:** Anthony Deally, Frauke Hackenberg, Grainne Lally, Matthias Tacke

**Affiliations:** Conway Institute of Biomolecular and Biomedical Research, Centre for Synthesis and Chemical Biology (CSCB), UCD School of Chemistry and Chemical Biology, University College Dublin, Belfield, Dublin 4, Ireland

## Abstract

Six new titanocene compounds have been isolated and characterised. These compounds were synthesised from their fulvene precursors using Super Hydride (LiBEt_3_H) followed by transmetallation with titanium tetrachloride to yield the corresponding titanocene dichloride derivatives. These complexes are bis-[((1-methyl-3-diethylaminomethyl)indol-2-yl)methylcyclopentadienyl] titanium (IV) dichloride **(5a)**, bis-[((5-methoxy-1-methyl,3-diethylaminomethyl)indol-2-yl)methylcyclopentadienyl] titanium (IV) dichloride **(5b)**, bis-[((1-methyl,3-diethylaminomethyl)indol-4-yl)methylcyclopentadienyl] titanium (IV) dichloride **(5c)**, bis-[((5-bromo-1-methyl)indol-3-yl)methylcyclopentadienyl] titanium (IV) dichloride **(5d)**, bis-[((5-chloro-1-methyl)indol-3-yl)methylcyclopentadienyl] titanium (IV) dichloride **(5e)**, and bis-[((5-fluoro-1-methyl)indol-3-yl)methylcyclopentadienyl] titanium (IV) dichloride **(5f)**. All six titanocenes **5a–5f** were tested for their cytotoxicity through MTT-based *in vitro* tests on CAKI-1 cell lines using DMSO and Soluphor P as solubilising agents in order to determine their IC_50_ values. Titanocenes **5a–5f** were found to have IC_50_ values of 10 (±2), 21 (±3), 29 (±4), 140 (±6), and 450 (±10) *μ*M when tested using DMSO.

## 1. Introduction

 Titanium-based reagents have been investigated for their use as anticancer agents for more than a decade and have been shown to have significant potential against solid tumours. In fact, budotitane ([cis-diethoxybis(1-phenylbutane-1,3-dionato)titanium (IV)]) which was designed by following cisplatin models was the first nonplatinum drug to enter clinical trials in 1993 [1]. Following on from this work was the use of titanocene dichloride as an anticancer compound in the clinic. Pioneering work by Köpf and Köpf-Maier cannot be overlooked as it was them who first identified the antitumor potential of metallocene dihalides [2]. The compound looked very promising during its preclinical evaluation; studies showed that an Ehrlich ascites cure of 100% and Colon 38 adenocarcinoma inhibition was better than that of cisplatin [3]. However, the use of titanocene dichloride as a single agent in the clinic was not sufficiently promising to warrant further studies, and titanocene dichloride has been discontinued from further clinical trials [4, 5]. One of the main downfalls of titanocene dichloride being used in the clinic was the uncertainty regarding the composition of the biologically active titanium species responsible for the antitumor activity. The hydrolysis products of titanocene dichloride at physiological pH have hampered identification of the active species responsible for antitumour activity [6]. 

Over the past decade, there has been an effort to prevent cross-resistance and increase the cytotoxicity of titanocene dichloride. This can be done using two distinct approaches, by either replacing the substituent at the cyclopentadienyl (Cp) ring or by anion exchange of the chlorines [7, 8]. The more investigated of these two routes is the replacement of the Cp rings with more elaborately substituted analogues. Gao et al. have synthesised steroidal-functionalised titanocene dichloride derivatives which could target hormone-dependant cancers such as prostate and breast cancer [9]. Recently, an effort was made by Zagermann et al. to synthesise titanocenes containing targeting substituents so as to increase selective uptake into cancer cells, and therefore reducing toxicity [10]. Titanocenes containing peptide mimics were synthesised; however, incorporation of the tumour-targeting oligopeptide enkephalin proved difficult under the conditions used. Nevertheless, it has been shown that it is principally possible to attach peptides to the titanocene, which may pave the way to tumour specific targeted titanocenes [10]. Allen et al. overcame the solubility issues of titanocene dichloride by incorporation of alkyl ammonium salts to the Cp ring to produce water soluble derivatives of titanocene dichloride [11]. These compounds were shown to have significantly better activity and stability than their nonfunctionalized counterpart titanocene dichloride and gave a potent cytotoxic effect on cisplatin-resistant ovarian A2780 tumor cell lines. Titanocenes which have varying alkyl ammonium chains have also been reported, this study showed that titanocenes could have nanomolar activity when substituted with diethylaminomethyl pendant chains [12].

We recently reported the use of indoles for the synthesis of both chiral and achiral titanocenes [13, 14]. Chiral titanocenes were shown to have lower activity when compared to their achiral counterparts; however, the IC_50_ values for these compounds may not be a true reflection of their cytotoxic activity as the chiral mixtures could not be separated. Following on from this work we decided to focus our attention on achiral titanocenes as these proved to be more active than their chiral counterparts. Substitution of the indole with a dimethylamino methyl arm and subsequent protonation of the amine with HCl gave the hydrochloride salt bis[(1-methyl-3-dimethylaminomethyl)indol-2-yl)methylcyclopentadienyl] titanium (IV) dichloride **1b**. This compound was tested on the renal cell carcinoma cell line CAKI-1 to give an IC_50_ value of 13 *μ*M when tested without any solubilising agent, and 0.71 *μ*M when tested with the solubilising agent soluphor P [15]. Also investigated by our group was varying the position of substitution of the indole to the Cp ring [16]. This study confirmed that substitution of the indole at position 4 warranted further investigation along with that of position 2.

Herein, we report the synthesis and cytotoxicity of novel achiral indole-substituted titanocene dichloride derivatives, which explore subtle structural changes to **1b**.

## 2. Results and Discussion

### 2.1. Synthesis

5-Fluoro-1H-indole, 5-chloro-1H-indole, 5-bromo-1H-indole, 4-bromo-1H-indole, and 5-methoxy-1H-indole were available commercially and used as received. The methylation of indoles was achieved by N-alkylation with NaH and methyliodide [17]. Introduction of an aldehyde group into indoles **3a–3c **(Figure 2) was achieved using *n*-BuLi followed by a DMF quench and subsequent workup in aq HCl [18]. As discussed earlier, we wished to incorporate the diethylaminomethyl moieties of benzyl-substituted titanocenes previously reported onto the indole, and this was done using the Mannich reaction [19]. Incorporation of the aldehyde group to position 3 of the indole was carried out using the Vilsmeier-Haack reaction with phosphorous oxychloride and DMF followed by a basic workup [20].

The synthesis of fulvenes **4a**–**4f** (Figure 3) from aldehydes **3a**–**3f** is based on a modified version of the Stone and Little method [21]. The aldehyde was reacted with freshly cracked cyclopentadiene in the presence of pyrrolidine as a base catalyst to yield the desired product in yields of 56–83%.

**Scheme 1:**
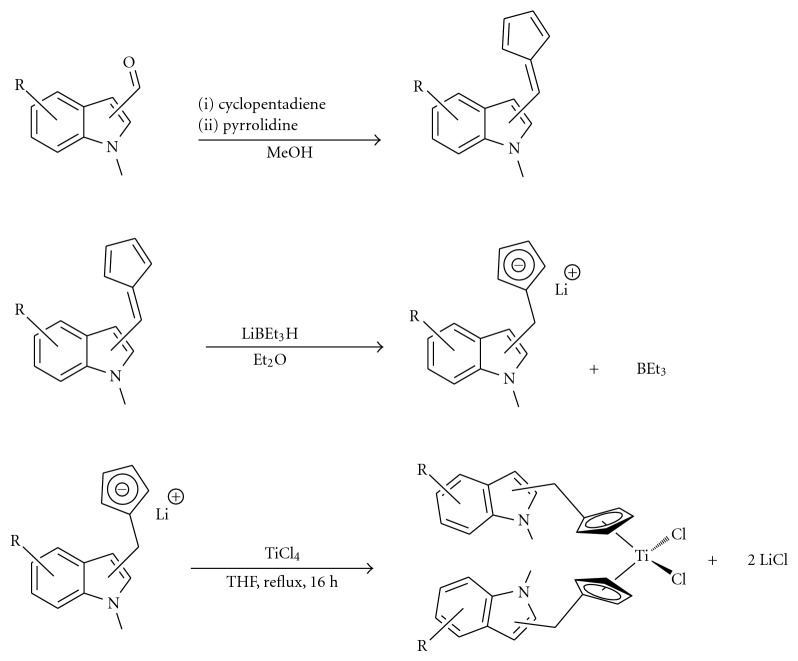
General reaction scheme for the synthesis of the fulvene precursors, and conversion to the corresponding indole-substituted titanocenes.

The exocyclic double bond in the fulvenes has increased polarity due to the inductive effects of its respective indole group. This allows for selective hydride insertion at this double bond and not at the diene component of the fulvenes. Hydride insertion was achieved using LiBEt_3_H (Superhydride) in diethyl ether to obtain the correspondingly functionalised lithium cyclopentadienide intermediate. With the exception of **5f**, these intermediates were then captured on a Schlenk glass frit, dried *in vacuo*, and redissolved in THF. Two equivalents of the lithium cyclopentadienide intermediate were then transmetallated with one equivalent of TiCl_4_, resulting in the formation of one equivalent of the appropriately substituted titanocenes **5a**–**5f** (Figure 4, Scheme 1) in yields of 30–85% and the byproduct lithium chloride, which was removed using celite. Repositioning of the Cp from position 2 of the indole to position 4 gave titanocenes in much higher yields (30% versus 85%) and higher purity. Titanocenes **5a**–**5c** were then treated with 2 equivalents of HCl in DCM/diethyl ether to produce the water soluble dihydrochloride salt of their diethylaminomethyl functionalised parent.

### 2.2. Cytotoxicity Studies

The cytotoxic effects of titanocenes **5a**–**5f** were determined using *in vitro* cultured human renal cell carcinoma CAKI-1 cells. The cell line was obtained from the ATCC (American Tissue Cell Culture Collection) (HTB-47) in 2001 and maintained in Dulbecco's modified eagle medium containing 10% (*v/v*) FBS (foetal bovine serum), 1% (*v/v*) penicillin streptomycin, and 1% (*v/v*) L-glutamine. Cytotoxicities of the various titanocenes were determined using MTT-based assays.

**Figure 1:**
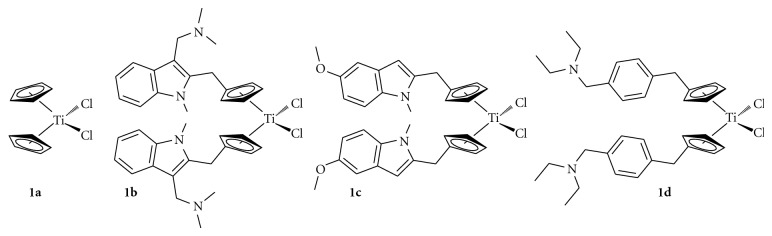
Structures of titanocene dichloride and previously synthesised indole-substituted titanocenes.

**Figure 2:**
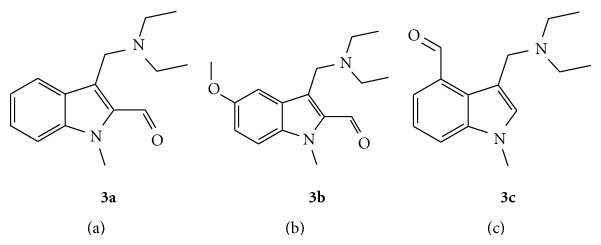
The structures of aldehydes **3a**–**3c** and the precursors to fulvenes** 4a**–**4c**.

**Figure 3:**
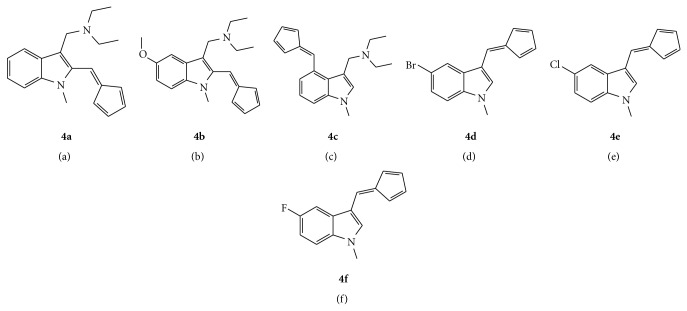
The structures of fulvenes **4a**–**4f**.

 This series described comprises of a range of titanocenes which vary greatly in their cytotoxicity. As can be seen in Table 1, Compounds **5a**–**5c **have moderate to high activity on the CAKI-1 cell line (Figures 5–7) whereas compounds **5d**–**5f **had little to no activity at all (Figure 8). This is very surprising as the introduction of halogens and in particular fluorine to medicinal compound classes has been shown to lead to an enhancement of the pharmaceutical properties in comparison to the parent compound in many cases [22]. In fact, it would be expected that the fluorine-substituted titanocene would be more cytotoxic than its bromine-substituted counterpart; however, this is not the case. A trend can be seen through substitution at position 5 of the indole ring, with more electron withdrawing groups having lower activity. This is more evident when compared with **1c **(Figure 1), where an electron donating methoxy-substituent is present at position 5 of the indole. This has an effect on increasing the activity of the parent indole two-fold to give an IC_50_ value of 11 *μ*M, whereas incorporation of electron withdrawing groups renders the compound inactive (depicted in Figure 8).

**Figure 4:**
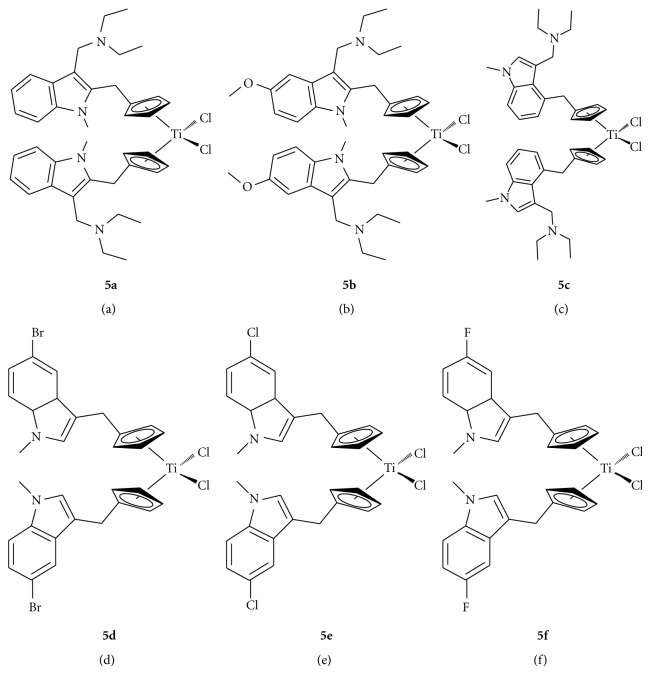
The structures of titanocenes **5a**–**5f**.

**Figure 5:**
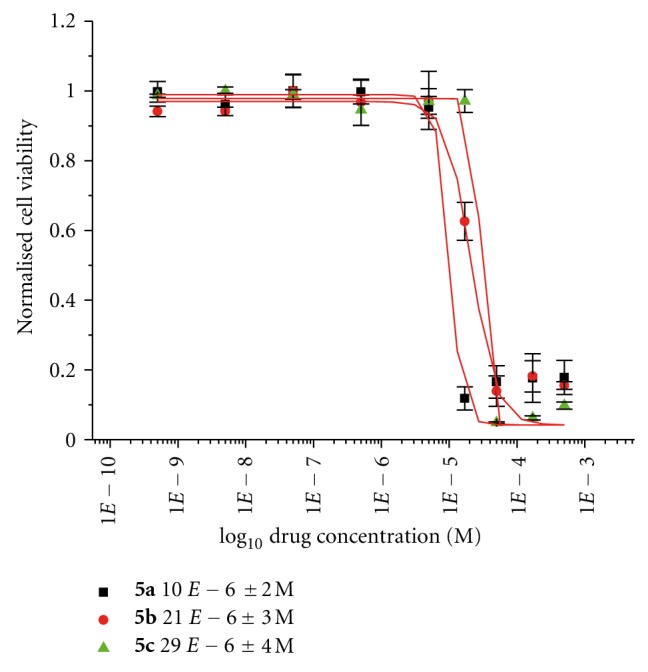
Cytotoxicity studies of titanocenes **5a**–**5c** against CAKI-1 cells using DMSO as cosolvent.

 As can be seen in Figure 4, we decided to incorporate the substitution pattern of the diethylaminomethyl-substituted titanocene **1d **with indole at position 3 to give titanocenes **5a** and **5c** as well as integrating this with the substitution pattern of **1b** to give compound **5b**. All three titanocenes were transformed to their hydrochloride salts to give them better solubility in biological medium. The compounds were tested with and without DMSO, which had a drastic effect on titanocene **5b** from 27 *μ*M (DMSO, Figure 5) to 75 *μ*M (no DMSO, Figure 7). The same trend can be seen for compound **5a** which is also bound to the Cp ring at position 2 of the indole ring. The opposite can be seen for compound **5c** which is bound to the Cp ring at position 4 of the indole. In this titanocene, the addition of DMSO decreases the activity of the compound.

**Figure 6:**
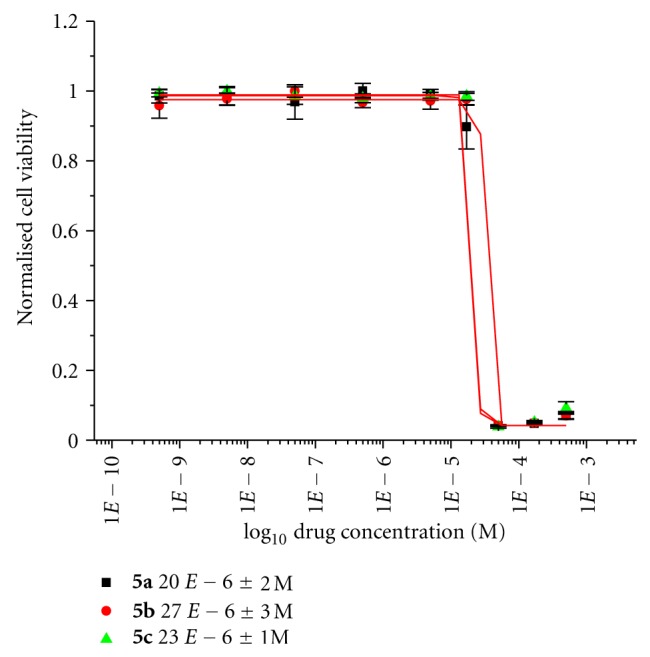
Cytotoxicity studies of titanocenes **5a**–**5c** against CAKI-1 cells using soluphor P as cosolvent.

 With the exception of compounds **5d**–**5f** (which showed low activity), the titanocenes were further tested using the solubilising agent soluphor P (2-pyrrolidone) on the CAKI-1 cell line (Figure 6). Soluphor P is a low-molecular-weight cyclic amide which is water-miscible and liquid at room temperature with solubilising properties. The toxicity of soluphor P is attractively low; its oral LD50 in rats is 5000 mg/kg. In one study, it was concluded that it is a better co-solvent than commonly used pharmaceutical cosolvents such as propylene glycol or glycerine [23]. Compound **1a **was tested on the CAKI-1 cell line using soluphor P as the solubilising agent, the result of which was a ten-fold increase in cytotoxicity from 8.2 *μ*M to 0.71 *μ*M when tested with DMSO.

 Not surprisingly, the compounds had the same trend, when soluphor P was used as the solubilising agent. Evident in Figures 5 and 6, it can be seen that titanocenes **5a **and** 5b **gained in potency when tested with soluphor P and DMSO, whereas compound **5c** suffered a slight loss in activity when tested on the CAKI-1 cell line with these solubilising agents. However, it is more beneficial for the compound to be tested in the clinic without the need for a solubilising agent, and for this reason compound **5c **is more advantageous for use in the clinic.

## 3. Conclusion and Outlook

Six new titanocenes, all similar in structure to previous compounds which exhibit promising cytotoxic activities, have been synthesised and characterised. The six compounds have been assessed for their cytotoxicities against the human kidney cancer cell line CAKI-1. Three of these compounds have been tested with DMSO, and three other compounds have been tested with DMSO, soluphor P, and without DMSO. The compounds display a range of activity, from inactive titanocenes substituted with electron withdrawing groups to compounds which have medium cytotoxicity (29 *μ*M) when tested using DMSO and higher activity (14 *μ*M) when tested with no solubilising agent. This is of course a huge advantage for use in the clinic. The incorporation of the indol-4-yl group onto the Cp ring has a profound effect on the activity of the titanocene. Both the parent compounds (N-((4-(cyclopenta-2,4-dienylmethyl)-1-methyl-1H-indol-3-yl)methyl)-N-ethylethanamine and titanocene dichloride) have much lower IC50 values when tested on their own with the indole component having no activity at all on the CAKI-1 cell line, whereas combining the two gives a 140 fold increase in activity to 14 *μ*M. It can be envisaged that this bioconjugate is helping to deliver the active species of the molecule to the cancer cell. However as with all anticancer agents the effect of the compound *in vitro* can be remarkably different than those *in vivo*, and for this reason we hope to test compound **5c **(referred to as Titanocene T from this paper on) in mice with xenografted CAKI-1 tumours in the near future.

## 4. Experimental

### 4.1. General Conditions

Titanium tetrachloride (1.0 M solution in toluene), Super Hydride (LiBEt_3_H, 1.0 M solution in THF), and all chemicals were obtained commercially from Aldrich Chemical Co. and used without any further purification. 1H-indole-4-carbaldehyde, 1H-indole-5-carbaldehyde and 6-bromo-1H-indole were purchased from Apollo Scientific and used as received. Solvents were dried in a Grubbs apparatus and collected under an atmosphere of nitrogen prior to use. All spectroscopic data of titanocenes **5a**–**5c** are of the hydrochloride salts of the parent compound. Manipulations of air and moisture sensitive compounds were done using standard Schlenk techniques, under a nitrogen atmosphere. NMR spectra were measured on a Varian 300, 400, or 500 MHz spectrometer. Chemical shifts are reported in ppm and are referenced to TMS. IR spectra were recorded on a Varian 3100 FT-IR Excalibur Series employing a KBr disk. UV-Vis spectra were recorded on a Cary 50 Scan UV-Visible Spectrophotometer, (*λ* in nm; *ε* in dm^3^ mol^−1 ^cm^−1^). The formulations were sonicated using a VWR Ultrasonic Cleaner (45 kHz). MS data was collected on a quadrupole tandem mass spectrometer (Quattro Micro, Micromass/Water's Corp., USA) and prepared as solutions of tetrahydrofuran (THF). CHN analysis was carried out with an Exeter-CE-440 elemental analyser, and Cl was determined by mercurimetric titrations.

### 4.2. MTT-Based Assay (MTT = 3-(4,5-Dimethylthiazol-2-yl)-2,5-diphenyl-2H-tetrazolium Bromide)

Preliminary *in-vitro* cell tests were performed on CAKI-1 cells to compare the cytotoxicity of the compounds presented in this work. The cell line was obtained from the ATCC (American Tissue Cell Culture Collection) (HTB-47) in 2001 and maintained in Dulbecco's modified eagle medium containing 10% (*v/v*) FBS (foetal bovine serum), 1% (*v/v*) penicillin streptomycin, and 1% (*v/v*) L-glutamine. The cytotoxicities of titanocenes **5a**–**5f** were determined by an MTT-based assay [24].

**Figure 7:**
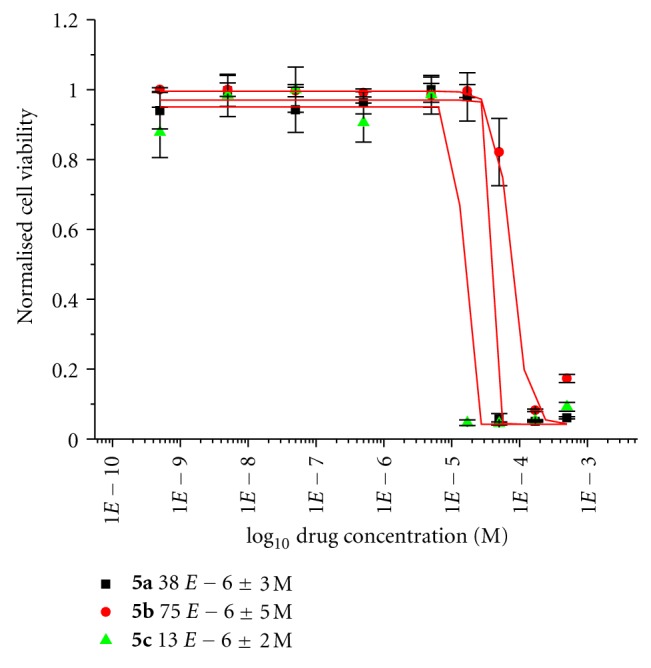
Cytotoxicity studies of titanocenes **5a**–**5c** against CAKI-1 cells without a cosolvent.

**Figure 8:**
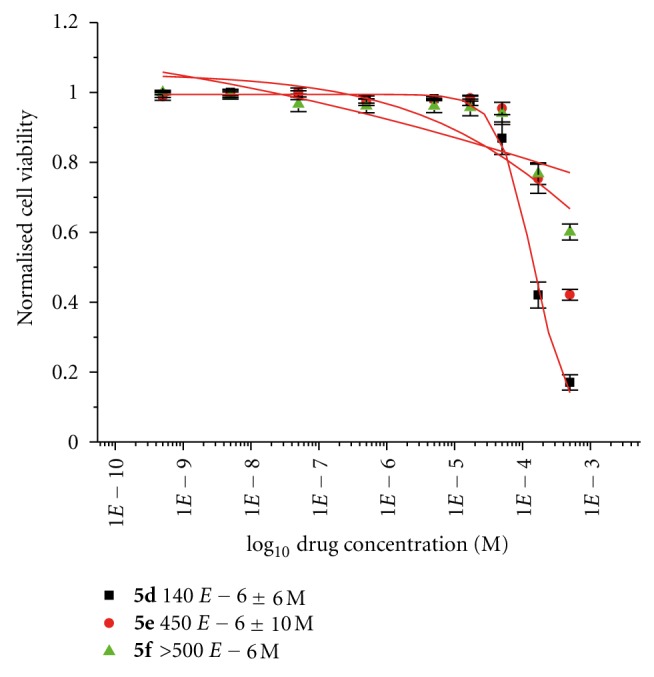
Cytotoxicity studies of titanocenes **5d**–**5f** against CAKI-1 cells using DMSO as co-solvent.

Specifically, cells were seeded in 96-well plates containing 200 *μ*L of medium and were incubated at 37°C and 5% carbon dioxide for 24 h to allow for exponential growth. The compounds used for testing were dissolved in dimethyl sulfoxide (70 *μ*L DMSO) and diluted with medium to obtain stock solutions of 5 × 10^−4 ^M in concentration. In the case of formulations using soluphor P, the compounds were dissolved in 50 *μ*L soluphor P and sonicated at room temperature for 5 min. The cells were then treated with varying concentrations of the compounds and incubated for 48 h at 37°C. At that time, the solutions were removed from the wells, the cells were washed with PBS (phosphate buffer solution), and fresh medium was added to the wells. Following a recovery period of 24 h incubation at 37°C, individual wells were treated with 200 *μ*L of a solution of MTT in medium (5 mg MTT per 11 mL medium). The cells were incubated for 3 h at 37°C. The medium was then removed, and the purple formazan crystals were dissolved in 200 *μ*L DMSO per well. Absorbance was then measured at 540 nm by a Wallac-Victor (Multilabel HTS Counter) plate reader or a SpectraMax 190 Microplate Reader. Cell viability was expressed as a percentage of the absorbance recorded for control wells. The values used for the dose response curves of Figures 5, 6, 7, and 8 represent the values obtained from four consistent MTT-based assays for each compound tested, and Titanocene Y (IC_50_ = 30 (±2) *μ*M) was used as a positive control in each test.

### 4.3. Synthesis

1-Methyl-3-diethylaminomethylindole was prepared as per the literature in 80% yield and verified via ^1^H NMR [25]. 

^1^H NMR (300 MHz, CDCl_3_)  *δ*  7.71 (*d*,  *J* = 7.9, 1H), 7.28 (*d*,  *J* = 8.1, 1H), 7.24–7.16 (*m*, 1H), 7.13–7.06 (*m*, 1H), 6.98 (*s*, 1H), 3.78 (*s*, 2H), 3.76 (*s*, 3H), 2.56 (*q*,  *J* = 7.1, 5H), 1.09 (*t*, *J* = 7.1, 7H).

**3-((Diethylamino)methyl)-1-methyl-1H-indole-2-carbaldehyde (3a)**

A solution of *n*-BuLi (5.0 mL, 12.5 mmol, and 2.5 M) was added to a Schlenk flask containing 1-methyl-3-diethylaminomethylindole (**2a**) (2.70 g, 12.5 mmol) dissolved in 30 mL THF at −78°C. The solution was warmed to 30°C and stirred at this temperature for 1 h, followed by an additional hour at room temperature. Then DMF (1.9 mL; 25 mmol) was added at −30°C and the reaction was allowed to come to room temperature over 24 h. The reaction was quenched by addition of 30 mL saturated ammonium chloride solution. This was extracted with DCM (2 × 50 mL). The organic layers were combined and washed with H_2_O (2 × 50 mL) and brine (2 × 50 mL). The DCM was dried over sodium sulfate, filtered and the solvent removed at reduced pressure to give a brown oil which was purified by column chromatography (silica) with DCM/pentane as the eluent to yield a brown oil in 85% yield (2.60 g; 10.6 mmol).

^1^H NMR (400 MHz, CDCl_3_)  *δ*  10.38 (*s*, 1H, CHO), 7.88 (*d*, *J* = 8.3 Hz, 1H), 7.40 (*t*,  *J* = 7.2 Hz, 1H), 7.34 (*d*,  *J* = 8.3 Hz, 1H), 7.15 (*t*,  *J* = 7.4 Hz, 1H), 4.07 (*s*, 3H, NCH_3_), 3.99 (*s*, 2H, CH_2_), 2.55 (*q*,  *J* = 6.9 Hz, 4H, NCH_2_CH_3_), 1.06 (*t*,  *J* = 6.9 Hz, 6H, NCH_2_CH_3_).

^13^C NMR (101 MHz, CDCl_3_)  *δ*  183.62 (CHO), 139.71, 132.48, 127.93, 127.11, 122.25, 120.51, 118.18, 110.34, 47.09 (CH_2_), 47.00 (NCH_2_CH_3_), 31.98 (CH_3_), 12.03 (NCH_2_CH_3_).

ES-MS: *m/z *172 [M–NCH_2_CH_3_]^+^.

IR (KBr disk, cm^−1^): 3052, 2966, 2933, 2871, 2807, 1684, 1812, 1525, 1469, 1380, 1201, 1166, 1118, 1060, 883, 742.

**N-((2-(Cyclopenta-2, 4-dienylidenemethyl)-1-methyl-1H-indol-3-yl)methyl)-N-ethylethanamine (4a)**

3-((Diethylamino)methyl)-1-methyl-1H-indole-2-carbaldehyde (**3a**) (2.5 g, 10 mmol) was dissolved in MeOH (70 mL). Freshly cracked cyclopentadiene (0.85 mL; 10 mmol) was added to the solution followed by pyrrolidine (0.84 mL; 10 mmol), and the colour gradually changed from yellow to red. After 20 h, acetic acid (1 mL) was added to the reaction, and the product was extracted with DCM (2 × 50 mL). The organic layers were combined and washed with H_2_O (2 × 50 mL) and brine (2 × 50 mL). The DCM was dried over sodium sulphate, and the solvent removed at reduced pressure to give a red oil, which was purified by column chromatography (silica) with DCM as the eluent to yield a red oil in 83% yield. (2.51 g; 8.56 mmol).

^1^H NMR (400 MHz, CDCl_3_)  *δ*  7.89 (*t*,  *J* = 7.3, 1H), 7.34–7.25 (*m*, 3H), 7.12 (*t*,  *J* = 7.3, 1H), 6.60 (*d*,  *J* = 5.2, 1H), 6.53 (*d*,  *J* = 5.2, 1H), 6.37 (*d*,  *J* = 5.2, 1H), 6.27 (*d*,  *J* = 5.3, 1H), 3.72 (*s*, 1H), 3.70 (*s*, 3H), 2.46 (*q*,  *J* = 7.1, 4H), 1.03–0.94 (*m*, 6H).

^13^C NMR (101 MHz, CDCl_3_)  *δ*  139.50, 134.50, 132.26, 131.63, 128.44, 127.78, 126.89, 126.07, 125.65, 123.15, 122.04, 120.95, 120.29, 110.12, 48.12, 46.80, 31.77, 11.84.

*λ*_max_ [nm], (*ε*) [L mol^−1 ^cm^−1^], CHCl_3_: 262 (13200), 388 (9500).

ES-MS: *m/z *220 [M–NCH_2_CH_3_]^+^.

IR (KBr disk, cm^−1^): 2940, 2856, 2808, 2756, 1634, 1611, 1545, 1456, 1419, 1340, 1315, 1255, 1202, 1044, 1075, 1009, 889, 771, 761.

**Dihydrochloride Derivative of bis-[((1-methyl-3-diethylaminomethyl)indol-2-yl)methylcyclopentadienyl] titanium (IV) dichloride (5a)**

Super Hydride (LiBEt_3_H) (8.6 mL, 8.6 mmol, and 1 M) in THF was concentrated by removal of the solvent by heating it to 60°C under reduced pressure of 10^−2^ mbar for 40 min and then to 90°C for 20 min in a Schlenk flask. N-((2-(cyclopenta-2,4-dienylidenemethyl)-1-methyl-1H-indol-3-yl)methyl)-N-ethylethanamine (**4a**) (2.5 g; 8.6 mmol) was added to a Schlenk flask and dissolved in dry diethyl ether (100 mL) to give a red solution. The red fulvene solution was transferred to the Super Hydride solution *via cannula*. The solution was left to stir for 16 h, in which time a yellow precipitate of the lithium cyclopentadienide intermediate formed, and the solution had changed its colour from red to white. The precipitate was filtered on to a frit. The white precipitate was dried briefly under reduced pressure and was transferred to a Schlenk flask under nitrogen. The lithium cyclopentadienide intermediate was dissolved in dry THF (50 mL) to give a pale yellow solution. Titanium tetrachloride (1.0 mL; 1.0 mmol) was added to the lithium cyclopentadienide intermediate solution to give a dark red solution. The dark red titanium solution was stirred for 16 h. The solvent was then removed under reduced pressure. The remaining residue was extracted with DCM (100 mL) and filtered through celite to remove the remaining LiCl. The solvent was removed under reduced pressure to yield an orange solid in 30% yield (1.00 g; 1.42 mmol). A portion of this solid (0.10 g; 0.14 mmol) was then dissolved in DCM (10 mL), an ethereal solution of hydrogen chloride (0.14 mL; 2 M) was added and a precipitate immediately formed. Diethyl ether (30 mL) was then added. After 15 min stirring, the solid was filtered to give a brown powder in 90% yield (0.10 g; 0.13 mmol).

^1^H NMR (400 MHz, DMSO)  *δ*  10.30 (*s*, 2H, NHCH_2_CH_3_), 7.77 (*d*,  *J* = 7.8 Hz, 2H), 7.43 (*d*,  *J* = 7.8 Hz, 2H), 7.17 (*t*,  *J* = 7.4 Hz, 2H), 7.11 (*t*,  *J* = 7.4 Hz, 2H), 6.77 (*s*, 4H, C_5_H_4_), 6.45 (*s*, 4H, C_5_H_4_), 4.47 (*s*, 4H,), 4.46 (*s*, 4H), 3.63 (*s*, 6H, NCH_3_), 3.09 (*s*, 8H, NCH_2_CH_3_), 1.29 (*t*,  *J* = 6.9, 12H, NCH_2_CH_3_).

^13^C NMR (101 MHz, DMSO)  *δ*  141.03, 136.98, 132.98, 127.90, 125.20 (C_5_H_4_), 122.09, 120.45, 119.03, 116.46 (C_5_H_4_), 110.37, 101.19, 46.90, 46.21 (NCH_2_CH_3_), 30.76 (NCH_3_), 26.47, 9.18 (NCH_2_CH_3_).

IR (KBr disk, cm^−1^): 3401, 2979, 2763, 1828, 1631, 1471, 1403, 1361, 1033, 894, 804, 746.

UV-Vis (CH_2_Cl_2_): *λ* 219 nm (*ε* 76,240), *λ* 282 nm (*ε* 23,666), *λ* 292 nm (*ε* 22,800), *λ* 400 nm (weak).

Microanalysis calculated for C_42_H_58_Cl_4_N_4_Ti (778.23 g/mol): Calcd. C, 62.38%; H, 7.23%; N, 6.93%; Found C, 62.56%; H, 7.98%; N, 6.41%.

5-Methoxy-1-methyl-1H-indole-2-carbaldehyde was prepared as per published procedure in 62% yield and verified by ^1^H NMR [26].

^1^H NMR (300 MHz, CDCl_3_)  *δ*  9.84 (*s*, 1H), 7.27 (*dd*,  *J* = 9.7, 6.7 Hz, 1H), 7.14 (*s*, 1H), 7.12–7.07 (*m*, 2H), 4.06 (*s*, 3H), 3.85 (*s*, 3H).

**3-((Diethylamino)methyl)-5-methoxy-1-methyl-1H-indole-2-carbaldehyde (3b)**

5-Methoxy-1-methyl-1H-indole-2-carbaldehyde (**2b**) (2.00 g, 10.5 mmol) was dissolved in acetic acid (40 mL), diethylamine (2.2 mL; 21 mmol) was added, and the solution was cooled to 30°C. Formaldehyde (2.3 mL, 21 mmol, and 30% in H_2_O) was added, and the solution was allowed to stand for 2 h. The mixture was then added to an ice/NaOH (100 mL; 9 M) solution and stirred vigorously for 10 min until a yellow precipitate formed. This was filtered, washed with water, and suction-dried to give a yellow solid in 72% yield (2.1 g; 7.7 mmol).

^1^H NMR (400 MHz, CDCl_3_)  *δ*  10.31 (*s*, 1H, CHO), 7.24 (*m*, 2H), 7.09 (*dd*,  *J* = 9.1, 2.5, 1H), 4.05 (*s*, 3H, OCH_3_), 3.95 (*s*, 2H, CH_2_), 3.87 (*s*, 3H, NCH_3_), 2.55 (*q*,  *J* = 7.1, 4H, NCH_2_CH_3_), 1.06 (*t*,  *J* = 7.1, 6H NCH_2_CH_3_).

^13^C NMR (101 MHz, CDCl_3_)  *δ*  183.05, 154.31, 135.25, 132.54, 127.00, 126.75, 118.85, 111.11, 101.68, 55.66 (CH_2_), 47.03, 46.75 (NCH_2_CH_3_), 31.87(NCH_3_), 11.91(NCH_2_CH_3_).

ES-MS: *m/z *202 [M–NCH_2_CH_3_]^+^.

IR (KBr disk, cm^−1^): 2964, 2831, 1660, 1523, 1490, 1461, 1384, 1238, 1168, 1043, 883, 802, 754.

**N-((2-(Cyclopenta-2,4-dienylidenemethyl)-5-methoxy-1-methyl-1H-indol-3-yl)methyl)-N-ethylethanamine (4b)**

3-((Diethylamino)methyl)-5-methoxy-1-methyl-1H-indole-2-carbaldehyde (**3b**) (1.5 g; 5.5 mmol) was dissolved in MeOH (70 mL). Freshly cracked cyclopentadiene (0.46 mL; 5.5 mmol) was added to the solution followed by pyrrolidine (0.45 mL; 5.5 mmol), and the colour gradually changed from yellow to red. After 16 h, acetic acid (1 mL) was added the reaction, and the product was extracted with DCM (2 × 50 mL). The organic layers were combined and washed with H_2_O (2 × 50 mL) and brine (2 × 50 mL). The DCM was dried over sodium sulphate, and the solvent removed at reduced pressure to give a red oil which was purified by column chromatography (silica) with DCM as the eluent to yield a red oil in 60% yield. (1.05 g; 3.26 mmol).

^1^H NMR (400 MHz, CDCl_3_)  *δ*  7.39 (*d*,  *J* = 2.4, 1H), 7.30 (*s*, 1H), 7.27–7.26 (*m*,  *J* = 5.5, 1H), 6.95 (*d*,  *J* = 2.5, 1H), 6.61 (*s*, 1H), 6.51 (*s*, 1H), 6.37 (*d*,  *J* = 5.0, 1H), 6.27 (*d*,  *J* = 5.2, 1H), 4.05 (*s*, 2H), 3.95 (*s*, 1H), 3.87 (*s*, 3H), 2.47 (*q*,  *J* = 7.1, 7H), 1.01 (*t*,  *J* = 7.1, 9H).

^13^C NMR (101 MHz, CDCl_3_)  *δ*  146.77, 134.42, 131.47, 130.91, 128.74, 126.10, 125.68, 122.25, 118.85, 114.79, 113.75, 111.11, 110.17, 102.37, 55.82, 48.34, 46.61, 32.01, 11.91.

ES-MS: *m/z *250 [M–NCH_2_CH_3_]^+^.

*λ*_max_ [nm], (*ε*) [L mol^−1 ^cm^−1^], CHCl_3_: 270 (14100), 390 (9800).

IR (KBr disk, cm^−1^): 2933, 2852, 2809, 2762, 1624, 1600, 1553, 1449, 1419, 1340, 1315, 1255, 1202, 1075, 1044, 1075, 1009, 889, 771, 761.

**Dihydrochloride Derivative of bis-[((5-methoxy-1-methyl,3-diethylaminomethyl)indol-2-yl)methylcyclopentadienyl] Titanium (IV) Dichloride (5b)**

Super Hydride (LiBEt_3_H) (5.0 mL, 5.0 mmol, and 1 M) in THF was concentrated by removal of the solvent by heating it to 60°C under reduced pressure of 10^−2 ^mbar for 40 min and then to 90°C for 20 min in a Schlenk flask. N-((2-(cyclopenta-2,4-dienylidenemethyl)-5-methoxy-1-methyl-1H-indol-3-yl)methyl)-N-ethylethanamine (**4b**) (1.60 g; 5.00 mmol) was added to a Schlenk flask and dissolved in dry diethyl ether (100 mL) to give a red solution. The red fulvene solution was transferred to the Super Hydride solution *via cannula*. The solution was left to stir for 16 h, in which time a yellow precipitate of the lithium cyclopentadienide intermediate formed, and the solution had changed its colour from red to white. The precipitate was filtered on to a frit. The white precipitate was dried briefly under reduced pressure and was transferred to a Schlenk flask under nitrogen. The lithium cyclopentadienide intermediate was dissolved in dry THF (50 mL) to give a pale yellow solution. Titanium tetrachloride (0.85 mL; 0.85 mmol) was added to the lithium cyclopentadienide intermediate solution to give a dark red solution. The dark red titanium solution was stirred for 8 h. The solvent was then removed under reduced pressure. The remaining residue was extracted with DCM (100 mL) and filtered through celite to remove the remaining LiCl. The solvent was removed under reduced pressure to yield a brown solid in 79% yield (1.51 g; 1.97 mmol). A portion of this solid (0.10 g; 0.13 mmol) was then dissolved in DCM (10 mL), and an ethereal solution of hydrogen chloride (0.14 mL; 2 M) was added, and a precipitate immediately formed. Diethyl ether (30 mL) was then added. After 15 min stirring, the solid was filtered to give a brown powder in 90% yield (0.10 g; 0.13 mmol).

^1^H NMR (300 MHz, DMSO)  *δ*  10.13 (*s*, 2H, NH), 7.41 (*m*, 4H), 6.89 (*s*, 2H), 6.43 (*s*, 4H, C_5_H_4_), 6.35 (*s*, 4H, C_5_H_4_), 4.44 (*s*, 4H), 4.16 (*s*, 4H), 3.88 (*s*, 6H, OCH_3_), 3.68 (*s*, 6H, NCH_3_), 3.16 (*s*, 8H, NCH_2_CH_3_), 1.32 (*d*,  *J* = 7.0, 12H, NCH_2_CH_3_).

^13^C NMR (75 MHz, DMSO)  *δ*  154.49, 148.64, 146.08, 141.85, 132.50 (C_5_H_4_), 132.04 (C_5_H_4_), 128.02, 111.45, 110.86, 101.48, 100.24, 56.04, 45.89, 43.49, 30.47 (NCH_3_), 26.57 (C_5_H_4_CH_2_), 9.05 (NCH_2_CH_3_).

IR (KBr disk, cm^−1^): 3491, 2981, 2362, 1828, 1621, 1486, 1234, 1162, 1037, 890, 835, 687.

UV-Vis (CH_2_Cl_2_): *λ* 223 nm (*ε* 83,450), *λ* 295 nm (*ε* 28,400), *λ* 400 nm (weak).

Microanalysis calculated for C_44_H_62_Cl_4_N_4_O_2_Ti (838.59 g/mol): C, 60.84%; H, 7.19%; N, 6.45%; found C, 60.12%; H, 7.42%; N, 6.34%.

**N-((4-Bromo-1-methyl-1H-indol-3-yl)methyl)-N-ethylethanamine (2c)**

4-Bromo-1-methyl-1H-indole (1.52 g, 7.15 mmol) was dissolved in acetic acid (40 mL), dimethylamine (1.5 mL; 14.3 mmol) was added, and the solution was cooled to 30°C. Formaldehyde (1.8 mL, 14.3 mmol, and 30% in H_2_O) was added, and the solution was allowed to stand for 2 h. The mixture was then added to an ice/NaOH (100 mL; 9 M) solution and stirred vigorously for 10 min until a yellow precipitate formed. This was filtered, washed with water, and suction-dried to give a yellow solid in 81% yield (1.71 g; 5.78 mmol).

^1^H NMR (400 MHz, CDCl_3_)  *δ*  7.24 (*d*,  *J* = 7.8, 1H), 7.21 (*d*,  *J* = 7.8, 1H), 7.12 (*s*, 1H, H-2), 7.00 (*t*,  *J* = 7.8, 1H, H-6), 4.08 (*s*, 2H, CH_2_), 3.73 (*s*, 3H, NCH_3_), 2.67 (*q*,  *J* = 6.7, 4H, NCH_2_CH_3_), 1.08 (*t*,  *J* = 7.1, 6H, NCH_2_CH_3_).

^13^C NMR (101 MHz, CDCl_3_)  *δ*  138.36, 129.57 (C-2), 128.29, 126.04, 123.45, 121.95 (C-6), 114.27, 108.42, 49.15 (CH_2_), 46.70 (NCH_2_CH_3_), 32.91 (NCH_3_), 11.76 (NCH_2_CH_3_).

ES-MS: *m/z *222 [M–NCH_2_CH_3_]^+^.

IR (KBr disk, cm^−1^): 2966, 2929, 2869, 2807, 2360, 1658, 1550, 1454, 1415, 1313, 1195, 1066, 838, 765.

**3-((Diethylamino)methyl)-1-methyl-1H-indole-4-carbaldehyde (3c)**

A solution of *n*-BuLi (2.3 mL, 5.8 mmol, and 2.5 M) was added to a Schlenk flask containing N-((4-bromo-1-methyl-1H-indol-3-yl)methyl)-N-ethylethanamine (**2c**) (1.7 g; 5.8 mmol) dissolved in 30 mL THF at −78°C. The solution was warmed to 30°C and stirred at this temperature for 1 h, followed by an additional hour at room temperature. Then DMF (0.9 mL; 12 mmol) was added at −30°C, and the reaction was allowed to come to room temperature over 24 h. The reaction was quenched by addition of 30 mL saturated ammonium chloride solution. This was extracted with DCM (2 × 50 mL). The organic layers were combined and washed with H_2_O (2 × 50 mL) and brine (2 × 50 mL). The DCM was dried over sodium sulfate, filtered, and the solvent removed at reduced pressure to give a brown oil. This was purified by acidic extraction to give a brown oil in 85% yield (1.41 g; 5.77 mmol).

^1^H NMR (400 MHz, CDCl_3_)  *δ*  10.80 (*s*, 1H, CHO), 7.79 (*d*,  *J* = 7.8, 1H), 7.52 (*d*,  *J* = 7.8, 1H), 7.30 (*t*,  *J* = 7.9, 1H, H-6), 7.15 (*s*, 1H, H-2), 3.82 (*s*, 2H, CH_2_), 3.81 (*d*, 3H, NCH_3_), 2.56 (*q*,  *J* = 6.9, 4H, NCH_2_CH_3_), 1.00 (*t*,  *J* = 6.6, 6H, NCH_2_CH_3_).

^13^C NMR (101 MHz, CDCl_3_)  *δ*  194.95 (CHO), 138.93, 131.67 (C-2), 130.27, 127.40, 121.03 (C-6), 120.19, 114.77, 113.25, 51.31(CH_2_), 45.85 (NCH_2_CH_3_), 32.86 (NCH_3_), 11.08 (NCH_2_CH_3_).

ES-MS: *m/z *172 [M–NCH_2_CH_3_]^+^.

IR (KBr disk, cm^−1^): 2967, 2933, 2804, 1671, 1608, 1550, 1456, 1402, 1317, 1199, 1054, 929, 765.

**N-((4-(Cyclopenta-2,4-dienylidenemethyl)-1-methyl-1H-indol-3-yl)methyl)-N-ethylethanamine (4c)**

3-((Diethylamino)methyl)-1-methyl-1H-indole-4-carbaldehyde (**3c**) (0.80 g; 3.3 mmol) was dissolved in MeOH (70 mL). Freshly cracked cyclopentadiene (0.27 mL; 3.3 mmol) was added to the solution followed by pyrrolidine (0.27 mL; 3.3 mmol), and the colour gradually changed from yellow to red. After 2 h, acetic acid (1 mL) was added the reaction, and the product was extracted with DCM (2 × 50 mL). The organic layers were combined and washed with H_2_O (2 × 50 mL) and brine (2 × 50 mL). The DCM was dried over sodium sulphate, and the solvent removed at reduced pressure to give a red oil in 82% yield, which could be used without further purification. (0.80 g; 2.72 mmol).

^1^H NMR (400 MHz, CDCl_3_)  *δ*  8.61 (*s*, 1H), 7.30 (*dt*,  *J* = 20.6, 6.6, 3H), 6.99 (*s*, 1H), 6.69 (*d*,  *J* = 5.2, 1H), 6.66–6.60 (*m*, 1H), 6.55 (*d*,  *J* = 5.1, 1H), 6.44 (*d*,  *J* = 5.1, 1H), 3.77 (*s*, 3H), 3.66 (*s*, 2H), 2.70–2.56 (*m*, 4H), 1.05 (*t*,  *J* = 7.1, 6H).

^13^C NMR (101 MHz, CDCl_3_)  *δ*  144.77, 140.42, 136.47, 133.11, 129.74, 129.10, 128.78 126.58, 125.27, 122.84, 120.89, 120.56, 112.65, 109.31, 55.81 (CH_2_), 46.62 (NCH_2_CH_3_), 32.21 (NCH_3_), 11.93 (NCH_2_CH_3_).

ES-MS: *m/z *220 [M–NCH_2_CH_3_]^+^.

*λ*_max_ [nm], (*ε*) [L mol^−1 ^cm^−1^], CHCl_3_: 280 (14500), 370 (8900).

IR (KBr disk, cm^−1^): 2933, 2852, 2809, 2762, 1624, 1600, 1553, 1449, 1419, 1340, 1315, 1255, 1202, 1075, 1044, 1075, 1009, 889, 771, 761.

**Dihydrochloride Derivative of bis-[((1-Methyl,3-diethylaminomethyl)indol-4-yl)methylcyclopentadienyl] Titanium (IV) Dichloride (5c)**

Super Hydride (LiBEt_3_H) (3.6 mL, 3.6 mmol, and 1 M) in THF was concentrated by removal of the solvent by heating it to 60°C under reduced pressure of 10^−2 ^mbar for 40 min and then to 90°C for 20 min in a Schlenk flask. N-((4-(cyclopenta-2,4-dienylidenemethyl)-1-methyl-1H-indol-3-yl)methyl)-N-ethylethanamine (**4c**) (1.06 g; 3.60 mmol) was added to a Schlenk flask and dissolved in dry diethyl ether (100 mL) to give a red solution. The red fulvene solution was transferred to the Super Hydride solution *via cannula*. The solution was left to stir for 16 h, in which time a yellow precipitate of the lithium cyclopentadienide intermediate formed, and the solution had changed its colour from red to white. The precipitate was filtered on to a frit. The white precipitate was dried briefly under reduced pressure and was transferred to a Schlenk flask under nitrogen. The lithium cyclopentadienide intermediate was dissolved in dry THF (50 mL) to give a pale yellow solution. Titanium tetrachloride (0.45 mL; 0.45 mmol) was added to the lithium cyclopentadienide intermediate solution to give a dark red solution. The dark red titanium solution was stirred for 8 h. The solvent was then removed under reduced pressure. The remaining residue was extracted with DCM (100 mL) and filtered through celite to remove the remaining LiCl. The solvent was removed under reduced pressure to yield a brown solid in 85% yield (1.08 g; 1.52 mmol). A portion of this solid (0.10 g; 0.14 mmol) was then dissolved in DCM (10 mL), and an ethereal solution of hydrogen chloride (0.14 mL; 2 M) was added, and a precipitate immediately formed. Diethyl ether (30 mL) was then added. After 15 min stirring, the solid was filtered to give a brown powder in 85% yield (0.090 g; 0.11 mmol).

^1^H NMR (400 MHz, D_2_O)  *δ*  7.34 (*s*, 2H, H-2), 7.27 (*d*,  *J* = 8.0, 2H), 6.98 (*t*,  *J* = 7.8, 2H, H-6), 6.47 (*d*,  *J* = 8.0, 2H), 6.01 (*s*, 4H, C_5_H_4_), 5.92 (*s*, 4H, C_5_H_4_), 4.00 (*s*, 4H), 3.95 (*s*, 4H, CH_2_), 3.66 (*s*, 6H, NCH_3_), 3.09–2.90 (*m*,  *J* = 7.0, 8H, NCH_2_CH_3_), 1.11–0.99 (*m*, 12H, NCH_2_CH_3_).

^13^C NMR (101 MHz, D_2_O)  *δ*  137.71, 137.54, 133.57 (C-2), 129.53, 124.73, 122.48 (C-6), 122.19, 118.24 (C_5_H_4_), 115.81 (C_5_H_4_), 109.68, 101.46, 49.24 (CH_2_), 46.10 (NCH_2_CH_3_), 33.27 (C_5_H_4_CH_2_), 32.57 (NCH_3_), 8.01 (NCH_2_CH_3_).

IR (KBr disk, cm^−1^): 3401, 2975, 2942, 2682, 2653, 2360, 1625, 1542, 1452, 1421, 1265, 1211, 985, 838, 752.

UV-Vis (CH_2_Cl_2_): *λ* 225 nm (*ε* 78,400), *λ* 290 nm (*ε* 27,500), *λ* 300 nm (*ε* 21,100), *λ* 400 nm (weak).

Micro analysis calculated for C_42_H_58_Cl_4_N_4_Ti (778.23 g/mol): Calcd. C, 62.38%; H, 7.23%; N, 6.93%; Found C, 61.96%; H, 7.47%; N, 6.75%.

5-Bromo-1-methyl-1H-indole-2-carbaldehyde was prepared as per literature in 90% yield and verified via ^1^H NMR [27].

^1^H NMR (300 MHz, CDCl_3_)  *δ*  9.9 (*s*, 1H), 8.46 (*s*, 1H), 7.66 (*s*, 1H), 7.44 (*dd*,  *J* = 8.5, 1H) 7.22 (*d*,  *J* = 7.2 1H) *d* 3.86 (3H, *s*).

**5-Bromo-2-(cyclopenta-2,4-dienylidenemethyl)-1-methyl-1H-indole (4d)**

5-Bromo-1-methyl-1H-indole-2-carbaldehyde (**3d**) (2.0 g; 8.4 mmol) was dissolved in MeOH (70 mL). Freshly cracked cyclopentadiene (0.71 mL; 8.4 mmol) was added to the solution followed by pyrrolidine (0.68 mL; 8.4 mmol), and the colour gradually changed from yellow to red. After 16 h an orange solid precipitated, this was filtered to give a 60% yield. (1.31 g; 4.54 mmol).

^1^H NMR (400 MHz, CDCl_3_)  *δ*  7.93 (*s*, 1H, H-4), 7.54 (*s*, 1H, H-2), 7.40–7.31 (*m*, 2H), 7.34 (*s*, 1H, C_5_H_4_C*H*), 7.20 (*d*,  *J* = 8.7, 1H), 6.71 (*s*, 1H, C_5_H_4_), 6.67–6.58 (*m*, 1H, C_5_H_4_), 6.46 (*s*, 1H,), 6.42–6.34 (*m*, 1H, C_5_H_4_), 3.84 (*s*, 3H, NCH_3_).

^13^C NMR (101 MHz, CDCl_3_)  *δ*  141.07, 135.67, 133.67 (C_5_H_4_), 132.22(C-2), 129.77 (C_5_H_4_CH), 129.06, 128.85 (C_5_H_4_), 126.91 (C_5_H_4_), 125.75, 121.71 (C-4), 121.68, 119.26 (C_5_H_4_), 114.57, 112.85, 111.21, 33.51 (NCH_3_).

*λ*_max_ [nm], (*ε*) [L mol^−1 ^cm^−1^], CHCl_3_: 275 (13000), 380 (9800).

ES-MS: *m/z *207 [M–Br + H]^−^.

IR (KBr disk, cm^−1^): 2923, 1612, 11527, 1454, 1390, 1338, 1292, 1240, 1078, 900.

**Bis-[((5-bromo-1-methyl)indol-3-yl)methylcyclopentadienyl] Titanium (IV) Dichloride (5d)**

Super Hydride (LiBEt_3_H) (2.8 mL, 2.8 mmol, and 1 M) in THF was concentrated by removal of the solvent by heating it to 60°C under reduced pressure of 10^−2 ^mbar for 40 min and then to 90°C for 20 min in a Schlenk flask. 5-Bromo-2-(cyclopenta-2,4-dienylidenemethyl)-1-methyl-1H-indole (**4d**) (0.80 g; 2.8 mmol) was added to a Schlenk flask and dissolved in dry diethyl ether (100 mL) to give a red solution. The red fulvene solution was transferred to the Super Hydride solution *via cannula*. The solution was left to stir for 16 h, in which time a light green precipitate of the lithium cyclopentadienide intermediate formed, and the solution had changed its colour from red to yellow. The precipitate was filtered on to a frit. The precipitate was dried briefly under reduced pressure and was transferred to a Schlenk flask under nitrogen. The lithium cyclopentadienide intermediate was dissolved in dry THF (50 mL) to give a pale green solution. Titanium tetrachloride (1.1 mL, 1.1 mmol, and 1 M) was added to the lithium cyclopentadienide intermediate solution to give a dark brown solution. The solution was stirred for 8 h. The solvent was then removed under reduced pressure. The remaining residue was extracted with DCM (100 mL) and filtered through celite to remove the remaining LiCl. The solvent was removed under reduced pressure to yield a dark green solid, which was washed with ether (50 mL) followed by pentane (50 mL) to give a green solid in 58% yield (0.56 g; 0.81 mmol).

^1^H NMR (400 MHz, CDCl_3_)  *δ*  7.62 (*s*, 2H, H-4), 7.32–7.25 (*m*, 2H, H-6), 7.21–7.09 (*m*, 2H, H-7), 6.88 (*s*, 2H, H-2), 6.37–6.22 (*m*, 8H, C_5_H_4_), 4.15 (*s*, 4H, CH_2_), 3.71 (*s*, 6H, NCH_3_).

^13^C NMR (101 MHz, CDCl_3_)  *δ*  137.72, 135.67, 129.32, 128.91 (C-2), 124.53 (C-6), 122.09 (C_5_H_4_), 121.53 (C*-*4), 116.02 (C_5_H_4_), 112.46, 111.95, 110.89 (C-7), 33.01 (NCH_3_), 26.43 (CH_2_).

IR (KBr disk, cm^−1^): 3114, 3012, 2925, 1475, 1435, 1375, 1214, 1045, 754.

UV-Vis (CH_2_Cl_2_): *λ* 219 nm (*ε* 80,100), *λ* 300 nm (*ε* 27,600), *λ* 410 nm (*ε* 4000).

Microanalysis calculated for C_30_H_26_Cl_2_N_2_Br_2_Ti (693.12 g/mol): Calcd. C, 51.99%; H, 3.78%; N, 3.94%; Found C, 52.48%; H, 3.89%; N, 3.84%.

5-Chloro-1-methyl-1H-indole-2-carbaldehyde was prepared as per the literature in 90% yield and verified via ^1^H NMR [28].

^1^H NMR (400 MHz, CDCl_3_)  *δ*  9.93 (*s*, 1H), 8.28 (*d*,  *J* = 1.4, 1H), 7.65 (*s*, 1H), 7.32–7.18 (*m*, 2H), 3.85 (*d*,  *J* = 6.0, 3H).

**5-Chloro-2-(cyclopenta-2,4-dienylidenemethyl)-1-methyl-1H-indole (4e)**

5-Chloro-1-methyl-1H-indole-2-carbaldehyde (**3e**) (2.60 g; 13.5 mmol) was dissolved in MeOH (70 mL). Freshly cracked cyclopentadiene (1.1 mL; 13.5 mmol) was added to the solution followed by pyrrolidine (1.1 mL; 13.5 mmol) and the colour gradually changed from yellow to red. After 16 h an orange solid precipitated, this was filtered to give a 56% yield. (1.81 g; 7.51 mmol).

^1^H NMR (400 MHz, CDCl_3_)  *δ*  7.78 (*s*, 1H, H-4), 7.57 (*s*, 1H, H-2), 7.35 (*s*, 1H, C_5_H_4_CH), 7.30–7.21 (*m*, 2H), 6.79–6.69 (*m*, 1H, C_5_H_4_), 6.69–6.60 (*m*, 1H, C_5_H_4_), 6.50–6.43 (*m*, 1H, C_5_H_4_), 6.43–6.34 (*m*, 1H, C_5_H_4_), 3.85 (*s*, 3H, NCH_3_).

^13^C NMR (101 MHz, CDCl_3_)  *δ*  140.92, 135.38, 133.64 (C_5_H_4_), 132.43 (C-2), 129.19 (C_5_H_4_CH), 128.81, 127.04 (C_5_H_4_), 126.90 (C_5_H_4_), 123.18 (C-4), 119.26 (C_5_H_4_), 118.61, 112.89, 110.82, 109.98, 33.57 (NCH_3_).

*λ*_max_ [nm], (*ε*) [L mol^−1 ^cm^−1^], CHCl_3_: 276 (12000), 370 (9000).

ES-MS: *m/z *207 [M–Cl + H]^−^.

IR (KBr disk, cm^−1^): 3018, 2400, 1616, 1529, 1475, 1438, 1357, 1340, 1214, 1081, 902, 769.

**Bis-[((5-chloro-1-methyl)indol-3-yl)methylcyclopentadienyl] Titanium (IV) Dichloride (5e)**

Super Hydride (LiBEt_3_H) (7.5 mL, 7.5 mmol, and 1 M) in THF was concentrated by removal of the solvent by heating it to 60°C under reduced pressure of 10^−2 ^mbar for 40 min and then to 90°C for 20 min in a Schlenk flask. 5-Chloro-2-(cyclopenta-2,4-dienylidenemethyl)-1-methyl-1H-indole (**4e**) (1.80 g, 7.50 mmol) was added to a Schlenk flask and dissolved in dry diethyl ether (100 mL) to give a red solution. The red fulvene solution was transferred to the Super Hydride solution *via cannula*. The solution was left to stir for 16 h, in which time a light green precipitate of the lithium cyclopentadienide intermediate formed, and the solution had changed its colour from red to yellow. The precipitate was filtered on to a frit. The precipitate was dried briefly under reduced pressure and was transferred to a Schlenk flask under nitrogen. The lithium cyclopentadienide intermediate was dissolved in dry THF (50 mL) to give a pale green solution. Titanium tetrachloride (2.4 mL, 2.4 mmol, and 1 M) was added to the lithium cyclopentadienide intermediate solution to give a dark brown solution. The solution was stirred for 8 h. The solvent was then removed under reduced pressure. The remaining residue was extracted with DCM (100 mL) and filtered through celite to remove the remaining LiCl. The solvent was removed under reduced pressure to yield a dark green solid, which was washed with ether (50 mL) followed by pentane (50 mL) to give a green solid in 84% yield (1.88 g; 3.12 mmol).

^1^H NMR (400 MHz, CDCl_3_)  *δ*  7.46 (*s*, 2H, H-4), 7.22–7.13 (*m*, 4H), 6.90 (*s*, 2H, H-2), 6.36–6.22 (*m*, 8H, C_5_H_4_), 4.15 (*s*, 4H, CH_2_), 3.71 (*s*, 6H, NCH_3_).

^13^C NMR (101 MHz, CDCl_3_)  *δ*  137.71, 135.41, 128.98 (C-2), 128.63, 124.90, 122.06 (C-6), 121.97 (C_5_H_4_), 118.43 (C*-*4), 115.94 (C_5_H_4_), 112.00, 110.40 (C-7), 32.90 (NCH_3_), 26.42 (CH_2_).

IR (KBr disk, cm^−1^): 3351, 3110, 2922, 1610, 1481, 1423, 1375, 1286, 1240, 1141, 1078, 1049, 833.

UV-Vis (CH_2_Cl_2_): *λ* 219 nm (*ε* 82,100), *λ* 310 nm (*ε* 26,100), *λ* 410 nm (*ε* 4500).

Microanalysis calculated for C_30_H_26_Cl_4_N_2_Ti (604.21 g/mol): Calcd. C, 59.63%; H, 4.34%; N, 4.64%; Found C, 58.12%; H, 4.40%; N, 4.24%.

5-Fluoro-1-methyl-1H-indole-2-carbaldehyde was prepared as per the literature in 90% yield and verified via ^1^H NMR [29].

^1^H NMR (400 MHz, CDCl_3_)  *δ*  9.95 (*s*, 1H), 7.97 (*dd*,  *J* = 9.2, 2.5, 1H), 7.68 (*s*, 1H), 7.29–7.19 (*m*, 1H), 7.08 (*td*,  *J* = 9.0, 2.5, 1H), 3.86 (*s*, 3H).

**5-Fluoro-2-(cyclopenta-2,4-dienylidenemethyl)-1-methyl-1H-indole (4f)**

5-Fluoro-1-methyl-1H-indole-2-carbaldehyde (**3f**) (2.0 g; 8.4 mmol) was dissolved in MeOH (70 mL). Freshly cracked cyclopentadiene (0.71 mL; 8.4 mmol) was added to the solution followed by pyrrolidine (0.68 mL; 8.4 mmol), and the colour gradually changed from yellow to red. After 16 h an orange solid precipitated, this was filtered to give a 60% yield (1.31 g; 4.54 mmol).

^1^H NMR (300 MHz, CDCl_3_)  *δ*  7.59 (*s*, 1H, (C_5_H_4_CH)), 7.45 (*d*,  *J* = 11.2, 1H), 7.35 (*s*, 1H), 7.22–7.26 (*m*, 1H), 7.04 (*t*,  *J* = 8.7, 1H), 6.73 (*s*, 1H), 6.64 (*s*, 1H), 6.46 (*s*, 1H), 6.40 (*s*, 1H), 3.86 (*s*, 3H).

^13^C NMR (75 MHz, CDCl_3_)  *δ*  161.03–159.62 (C*-*5), 133.46 (C_5_H_4_), 132.84 (C_5_H_4_CH), 130.42, 129.50, 128.63 (C_5_H_4_), 127.74, 127.63, 126.87 (C_5_H_4_), 119.26 (C_5_H_4_), 111.42–111.07, 110.64–110.51, 109.99, 104.41–104.09 (C-7), 33.62.

^19^F NMR (282 MHz, CDCl_3_)  *δ*  −122.50 (*td*,  *J* = 9.2, 4.2, 1F).

ES-MS: *m/z *207 [M–F + H]^−^.

*λ*_max _  [nm], (*ε*) [L mol^−1 ^cm^−1^], CHCl_3_: 262 (15400), 390 (1100).

IR (KBr disk, cm^−1^): 2933, 2852, 2809, 2762, 1624, 1600, 1553, 1449, 1419, 1340, 1315, 1255, 1202, 1075, 1044, 1075, 1009, 889, 771, 761.

**Bis-[((5-fluoro-1-methyl)indol-3-yl)methylcyclopentadienyl] Titanium (IV) Dichloride (5f)**

Super Hydride (LiBEt_3_H) (12.0 mL, 12.0 mmol, and 1 M) in THF was concentrated by removal of the solvent by heating it to 60°C under reduced pressure of 10^−2 ^mbar for 40 min and then to 90°C for 20 min in a Schlenk flask. 5-fluoro-2-(cyclopenta-2,4-dienylidenemethyl)-1-methyl-1H-indole (**4f**) (2.70 g; 12.0 mmol) was added to a Schlenk flask and dissolved in dry diethyl ether (100 mL) to give a red solution. The red fulvene solution was transferred to the Super Hydride solution *via cannula*. The solution was left to stir for 16 h, in which time a sticky dark green precipitate of the lithium cyclopentadienide intermediate formed, and the solution had changed its colour from red to yellow. The solution was removed using a syringe and the intermediate was washed with ether (30 mL). The lithium cyclopentadienide intermediate was dissolved in dry THF (50 mL) to give a pale green solution. Titanium tetrachloride (2.4 mL, 2.4 mmol, and 1 M) was added to the lithium cyclopentadienide intermediate solution to give a dark green solution. The solution was stirred for 8 h. The solvent was then removed under reduced pressure. The remaining residue was extracted with DCM (100 mL) and filtered through celite to remove the remaining LiCl. The solvent was removed under reduced pressure to yield a dark green solid which was washed with ether (50 mL) followed by pentane (50 mL) to give a green solid in 55% yield (1.90 g; 3.31 mmol).

^1^H NMR (400 MHz, CDCl_3_)  *δ*  7.22–7.09 (*m*, 4H), 7.00–6.87 (*m*, 4H), 6.33 (*s*, 4H, C_5_H_4_), 6.28 (*s*, 4H, C_5_H_4_), 4.14 (*s*, 4H, CH_2_), 3.71 (*s*, 6H, NCH_3_).

^13^C NMR (101 MHz, CDCl_3_)  *δ*  158.80–156.47 (C*-*5), 137.72, 133.66, 129.22, 128.21, 122.14 (C_5_H_4_), 115.94 (C_5_H_4_), 112.30–112.25, 110.17–110.05, 109.96–109.90, 103.98–103.75, 32.95 (NCH_3_), 26.55 (CH_2_).

^19^F NMR (282 MHz, CDCl_3_)  *δ* (−125.09)–(−125.19) (*m*, 1F).

UV-Vis (CH_2_Cl_2_): *λ* 225 nm (*ε* 85,200), *λ* 310 nm (*ε* 26,100), *λ* 410 nm (*ε* 4000).

Microanalysis calculated for C_30_H_26_Cl_2_N_2_F_2_Ti (571.31 g/mol): Calcd. C, 63.07%; H, 4.59%; N, 4.9%; Found C, 63.15%; H, 4.05%; N, 3.97%.

## Figures and Tables

**Table 1:** Cytotoxicities of titanocenes **5a**–**5f** on CAKI-1 cells using DMSO-, medium-, and soluphor P-based formulations.

Compound	IC50 [*μ*M] (DMSO)	IC50 [*μ*M] (pure medium)	IC50 [*μ*M] (soluphor P)
**5a**	10 (±2)	38 (±3)	20 (±2)
**5b**	21 (±3)	75 (±5)	27 (±3)
**5c**	29 (±4)	13 (±2)	23 (±1)
**5d**	140 (±6)	Not measured	Not measured
**5e**	450 (±10)	Not measured	Not measured
**5f**	>500	Not measured	Not measured
